# The Difference in Supine versus Standing Plain Radiograph of the Knee in Patients with Knee Osteoarthritis

**DOI:** 10.5704/MOJ.2507.007

**Published:** 2025-07

**Authors:** AA Musa, R Shamsuddin-Perisamy, CL Low, AH Zulkifly, RY Kow

**Affiliations:** 1 Department of Orthopaedics, Traumatology and Rehabilitation, International Islamic University Malaysia, Kuantan, Malaysia; 2 Department of Radiology, International Islamic University Malaysia, Kuantan, Malaysia

**Keywords:** knee osteoarthritis, standing supine radiograph

## Abstract

**Introduction:**

Knee osteoarthritis is a degenerative joint disease attributed to failure in joint repair process. Key aspect of the diagnosis relies on thorough history, along with physical examination and radiology findings. The conventional weight-bearing plain radiograph remains the key modality to determine the severity of the condition and helps to plan the surgery. Nevertheless, not all patients can undergo weight-bearing plain radiographs, especially those who are wheelchair-bound or have severe deformities. The purpose of this study is to investigate whether a weight-bearing plain radiograph of the knee is essential in all patients with knee osteoarthritis.

**Materials and Methods:**

A prospective cohort study on patients with knee osteoarthritis receiving treatment in a single tertiary hospital was conducted. All patients consented to participate in this study. Patients were assessed functionally with the Western Ontario and McMaster Universities Osteoarthritis Index (WOMAC) and radiologically with plain radiographs. Patients were subjected to undergo both supine and standing plain radiographs of the knee in the same setting for comparison purposes where measurement is done following patient functional outcome and radiological measurement for the patient.

**Results:**

Our study shows that reduction in joint space is more obvious in weight-bearing radiographs, however in severe or higher-grade osteoarthritis, a supine radiograph is adequate to diagnose knee osteoarthritis

**Conclusion:**

Standing radiograph of the knee is preferred to a supine knee radiograph wherever possible due to the additional value it brings, however, in certain patient conditions, a supine radiograph is still acceptable.

## Introduction

Knee osteoarthritis is a chronic and debilitating condition, and it is a progressive joint disease due to failure in the joint repair process^1-5^. It is well known that this condition has emerged as one of the highest causes of disability in musculoskeletal disease, especially in the elderly population. The diagnosis of knee osteoarthritis requires a thorough history taking, along with clinical examination, radiological findings, and blood investigations, to execute a solid plan of management and good pre-operative planning for this condition^2-6^.

One of the most important radiological assessments needed for diagnosing knee osteoarthritis is a plain radiograph, where hallmark features of osteoarthritis such as joint space narrowing, osteophyte, subchondral sclerosis, and bony deformity will be present^3-8^. Over the years, debate has arisen regarding the best radiography for detecting knee osteoarthritis^3-8^. A few options available include the supine anteroposterior (AP) and lateral view, or the lateral view along with standing or weight-bearing AP view. Guidelines have been suggesting that the weight-bearing AP view is useful in detecting knee osteoarthritis, as weight-bearing will further reveal the narrowing of joint space. Nevertheless, occasionally it is difficult to obtain the weight-bearing AP view due to difficulty in standing, not to forget the internal rotation of the tibia in extension and exaggeration of reduced joint space in the presence of knee ligament laxity. Despite the guideline suggestion of weight-bearing AP knee radiograph for diagnosis of osteoarthritis, this procedure proved difficult in some patients because of their pain and limitation^7^. Moreover, most government and private health clinics are not equipped with the facilities to perform a weight-bearing radiograph of the knee and whole lower leg. With the experience of the recent COVID-19 pandemic in mind, the question remains whether patients require a weight-bearing plain radiograph in the hospital setting to make the diagnosis of knee osteoarthritis^9-13^.

Hence, we aim to conduct a study to compare the significance of both standing and supine knee radiograph in aiding the diagnosis of osteoarthritis.

## Materials and Methods

We conducted a prospective cohort study of our osteoarthritis patient who were receiving treatment at our centre. All patients diagnosed with osteoarthritis were identified from the clinical visits and electronic record system, and their demographic, clinical and radiological data were extracted and reviewed. The diagnosis of Osteoarthritis was made based on clinical criteria alone, clinical and laboratory criteria or clinical and radiographic criteria, following the diagnostic guidelines for osteoarthritis outlined by the American College of Rheumatology 1986 criteria as suggested by the Malaysia Clinical Practice Guidelines for Osteoarthritis.

Patients diagnosed with osteoarthritis underwent radiological assessments, including supine and standing knee radiographs in anteroposterior (AP) view, lateral view, patellar skyline view, and whole-leg standing radiograph. These images were analysed to measure joint space and the tibiofemoral angle. Additionally, the functional status of knee osteoarthritis was evaluated using the WOMAC score.

The inclusion criteria for patient selection in this study were a diagnosis of knee osteoarthritis, the ability to stand and tolerate a standing AP knee radiograph, and a willingness to participate in the study. Patients who were unable to stand for the knee radiograph were excluded.

**Fig. 1:**
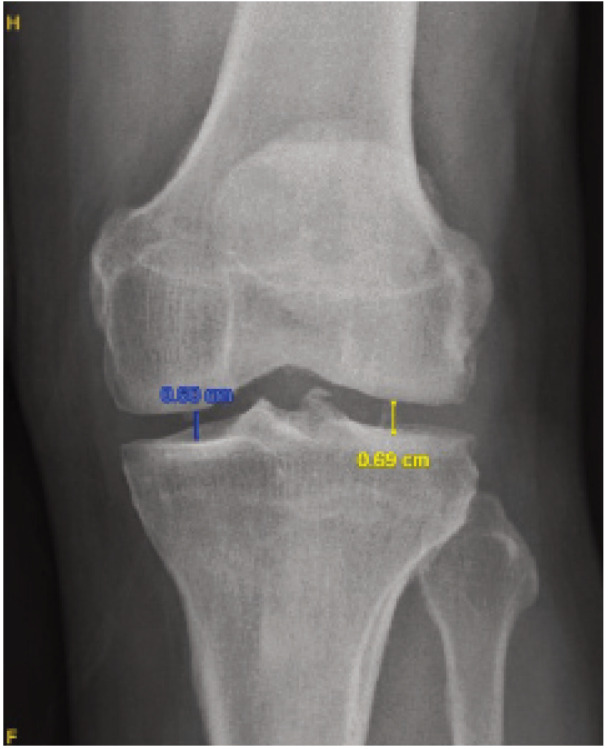
Joint space measurement method.

**Fig. 2:**
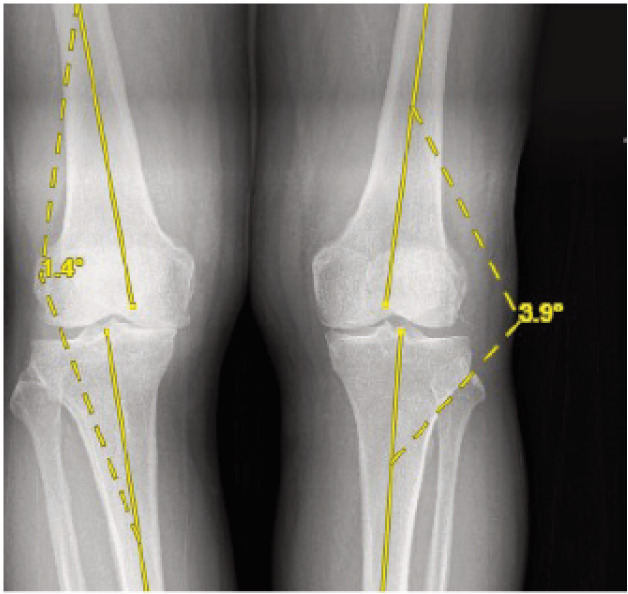
Tibiofemoral angle measurement.

Radiological measurements included the medial and lateral joint spaces in the knee’s AP view for both standing and supine radiographs, as well as the tibiofemoral angle from the whole-leg standing radiograph. The medial joint space was measured from the midpoint of the medial femoral condyle to the midpoint of the medial tibial condyle, while the lateral joint space was measured from the midpoint of the lateral femoral condyle to the midpoint of the lateral tibial condyle, as illustrated in Fig. 1. The tibiofemoral angle was determined by measuring the angle between the anatomical axes of the femur and tibia, as shown in Fig. 2. To enhance the accuracy of interpretation, measurements were independently performed by an orthopaedic medical officer and a radiologist, and the results were compared to validate the method.

The statistical significance of the tibiofemoral angle and the medial and lateral joint spaces was assessed based on the results obtained. Principal component analysis (PCA) was conducted using JASP version 0.173, a statistical software developed by the University of Amsterdam, to identify key factors contributing to knee osteoarthritis.

This study was conducted with the approval of the local research ethics committee, and all research activities adhered to Good Clinical Practice and the required ethical standards.

## Results

A total of 75 samples were included in the study, with a mean age of 66 years old with the youngest age group being 37 years old, and the oldest was 78 years old. The mean lateral joint space measurement in the supine knee was 52mm, ranging from a minimum of 18mm to a maximum of 82mm, with a standard deviation of 15mm. For the medial joint space in the supine position, the mean was 31mm, ranging from minimum of 3mm to a maximum of 59mm, with a standard deviation of 15mm. In standing radiographs, the mean joint space was 20mm for the medial side and 56mm for the lateral side. A reduction in joint space was noted in the medial compartment of the knee in standing compared to supine, with a difference of approximately 10mm (31mm to 20mm). Descriptive analysis of functional outcomes using the WOMAC score revealed a mean pain score of 8.9, a stiffness score of 3.4, a functional score of 32.6, and a mean total score of 45.1.

To validate the measurements, we obtained readings from both an orthopaedic trainee and a radiologist to compare the measurement differences. A correlation test was performed on these readings to assess their consistency. The correlation test indicated a significant correlation for both joint space and tibiofemoral angle measurements (P value < 0.01), confirming the accuracy of the inter-observer reliability test.

Following the validation of measurements, we analysed the correlation between the tibiofemoral angle and joint space. The results showed a significant correlation between the tibiofemoral angle and joint space in both the supine and standing groups, for both the medial and lateral sides (as shown in Table I).

**Table I:** Correlation between tibiofemoral angle to joint space.

Measure 1		Measure 2	t	df	p
TFA	-	Medial joint space supine	-13.690	61	< .001
TFA	-	Medial joint space standing	-8.652	61	< .001
TFA	-	Lateral joint space supine	-28.711	61	< .001
TFA	-	Lateral joint space standing	-24.658	61	< .001

Note: Student's t-test.

We then proceeded to determine the significance of either the medial or lateral joint space in relation to tibiofemoral angle and osteoarthritis. We employed a Paired Sample T-test to compare joint space measurements in both standing and supine radiographs. The analysis revealed that the medial joint space was significant, while the lateral joint space was not (as shown in Table II).

**Table II:** Correlation between joint space in supine and standing AP knee radiograph.

Measure 1		Measure 2	t	df	p
Medial joint space standing	-	Medial joint space supine	-7.891	74	< .001
Lateral joint space standing	-	Lateral joint space supine	2.599	74	0.011

Note: Student's t-test.

**Fig. 3:**
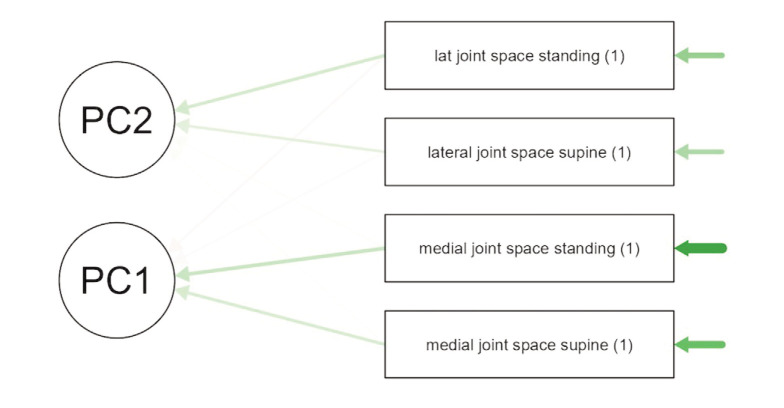
Principal component analysis.

Principal Component Analysis was subsequently conducted to ascertain the significance of the medial joint space in supine and standing radiographs. The analysis indicated that the medial joint space in supine and lateral radiographs is unique and holds a higher value in the principal component than the lateral joint space in supine and standing. This suggests that the medial joint space holds greater significance in osteoarthritis conditions, as demonstrated in Fig. 3 and Table III. Table IV shows that component 1 of the medial joint space in supine and standing also exhibits higher Eigenvalues compared to the lateral joint space in supine and standing, indicating that this component significantly contributes to and is important in determining knee osteoarthritis.

**Table III:** Principal component analysis of factor determining knee osteoarthritis.

	PC1	PC2	Uniqueness
Medial joint space supine	0.940	0.036	0.115
Medial joint space standing	0.940	0.004	0.117
Lateral joint space standing	-0.093	0.875	0.226
Lateral joint space supine	-0.070	0.879	0.223

Note: Applied rotation method is varimax.

**Table IV:** Component characteristic in principal component analysis.

	Eigenvalue	Proportion var.	Cumulative
Component 1	609.396	0.522	0.522
Component 2	378.896	0.324	0.846

Following the determination that the medial joint space is more significant than the lateral joint space, to establish which radiograph is more significant, whether the standing medial joint space or the supine medial joint space, we refer to Fig. 3 and Table III and Table IV. Here, we observe that standing medial joint space contributes more weight in determining osteoarthritis, followed by supine medial joint space. This conclusion is based on the values observed through the rotation method in principal component analysis, where we note the component loading from component 1 to component 2. In this comparison, the medial joint space in standing radiographs provides a lower value than the supine position, indicating that it is more significant.

On the other hand, the WOMAC score showed no significant correlation with osteoarthritis condition, joint space reduction, or high tibiofemoral angle results (as shown in Table V).

**Table V:** Correlation of WOMAC score and joint space reduction.

	Variable	WOMAC Total Score
WOMAC total score	Pearson's r	-
	p-value	-
Medial joint space supine	Pearson's r	- 0.059
	p-value	0.614
Lat joint space standing	Pearson's r	0.038
	p-value	0.743
Medial joint space standing	Pearson's r	-0.205
	p-value	0.078
Lateral joint space supine	Pearson's r	- 0.172
	p-value	0.139

Based on the results of our analysis, standing radiographs demonstrated greater reduction in joint space compared to supine knee radiographs. However, while weight-bearing radiographs offer added value in cases of doubtful joint space reduction in knee osteoarthritis, they are not necessary for diagnosing high-grade osteoarthritis. Symptomatic knee osteoarthritis or functional limitations showed no statistically significant correlation with the osteoarthritis condition.

## Discussion

Osteoarthritis is a chronic disease of failure in the knee joint repair process which comes from the thinning of joint cartilage along in reducing the thickness of synovial fluid hence causing the narrowing of joint space is more significant in standing radiographs compared to the supine radiograph^4,14^. This phenomenon also can be seen clearly in our study which shows the reduction in both medial and lateral joint surfaces in all samples involved. Upon weight-bearing condition, cartilaginous surfaces will impinge on each other thus making the bony surface closer together hence making deformity if present become more obvious.

In osteoarthritis radiological features, reduction of medial joint space is encountered more compared to the lateral joint space hence our analysis shows that the medial joint space reduction in both AP standing and supine radiograph to be significantly correlated with osteoarthritis with standing AP is more significant compared to the supine AP evidence by higher F value following ANOVA statistic test method.

Based on our findings, the reduction of medial joint space is a reliable indicator for diagnosing knee osteoarthritis in both supine and standing radiographs. This suggests that a supine radiograph may be sufficient when clinical features strongly suggest osteoarthritis, particularly in cases of severe pain or deformity where weight-bearing AP views are challenging to obtain. Similar limitations in obtaining standing radiographs were noted in studies by Johnson *et al* (1980)^15^ and Grelsalmer (1995)^16^. However, if a patient can tolerate standing radiographs, the standing AP view remains more beneficial for diagnosis and management. Pre-operative assessment of knee osteoarthritis depends on the degree of deformity.

The Hip-Knee-Ankle (HKA) alignment is considered the gold standard for assessing coronal plane deformity in knee osteoarthritis due to its association with load distribution within the joint. However, HKA assessment requires a long-leg standing radiograph, which may not be feasible for all patients due to the inability to stand, the need for specialised equipment, and higher radiation exposure. An alternative is tibiofemoral alignment, which can be assessed using standard knee radiographs and offers a more convenient and accessible approach^17^.

Since the knee is a load-bearing joint, the tibiofemoral angle often shows greater varus alignment in standing positions compared to supine positions due to the influence of soft tissue constraints. This observation aligns with findings by Brown *et al* (2019)^14^ and our study, which demonstrated similar results, including further reductions in medial joint space. Brouwer *et al* (2003)^4^ also reported an average of 2° greater varus deviation in patients without gross collateral ligament laxity.

Coronal plane alignment of the knee is a significant concern in managing knee osteoarthritis. Achieving ideal coronal alignment in knee arthroplasty remains a challenge due to evolving concepts such as anatomical alignment, mechanical alignment, and kinematic alignment. To better understand alignment deformities, long-leg radiographs are the preferred investigation. These allow for measurements of the Hip-Knee-Ankle (HKA) angle, mechanical proximal tibial angle, mechanical lateral distal femoral angle, arithmetic HKA, and joint line obliquity, which are essential for classifying coronal plane alignment^18^.

Based on coronal plane alignment classification, knee deformities can be categorised as varus, neutral, or valgus, with the apex of the deformity located distally, neutrally, or proximally. However, the classification of alignment differences is not the primary focus of this study.

The Functional score is beneficial in the assessment of the symptom and monitoring of the progression of functional limitation in the patient and aids in the management of the patient however from our study it does not seem to correlate with the degree of osteoarthritis in the patient and the degree of joint reduction.

However, there is a limitation in this study as this study was designed as a cross sectional study and has no control group of patients with normal knees in a similar age group as a controlled. Further studies may include radiographs in multiple views of radiograph such as AP weight bearing in full extension, 30° flexion, and 45° knee with controlled groups of the same degree of flexion in supine as some literature shows the variety of weight bearing in different angle aid in the diagnosis of osteoarthritis. Further study also can be done to evaluate the component of tibial rotation to determine the 3D pathology of the osteoarthritis condition.

## Conclusion

Standing AP knee radiograph is preferred if a single radiograph is to be taken for diagnosis of knee osteoarthritis as weight-bearing radiographs will exaggerate deformity and reduction of joint space in osteoarthritis patients however if there is a limitation in obtaining this radiograph, supine AP knee radiograph proved to be an adequate alternative.
